# Practice Does Not Make Perfect: The Tireless Pursuit of Achieving Perfect Sleep

**DOI:** 10.3390/ijerph18168523

**Published:** 2021-08-12

**Authors:** Natalie D. Dautovich, Morgan P. Reid, Sahar M. Sabet, Sarah M. Ghose, Joseph M. Dzierzewski

**Affiliations:** Psychology Department, Virginia Commonwealth University, P.O. Box 842018, Richmond, VA 23284-2018, USA; reidmp@vcu.edu (M.P.R.); sabetsm@vcu.edu (S.M.S.); ghosesm@vcu.edu (S.M.G.); dzierzewski@vcu.edu (J.M.D.)

**Keywords:** sleep health, perfectionism, dysfunctional beliefs about sleep, sleep competency, insomnia, college students

## Abstract

Objective: Perfectionism is consistently identified as a predisposing and perpetuating factor for a wide range of mental health conditions and disorders. Given the unique cognitive, emotional, and physiological characteristics associated with perfectionism, perfection could have serious implications for a critical health behavior—our sleep. The current study examines the links between perfectionism and sleep health with the goal of identifying potential sleep-related beliefs as underlying mechanisms. Methods: Participants were 417 undergraduate students at a large, public university in the mid-Atlantic United States. Participants completed a one-time online survey with the Almost Perfect Scale-Revised, the Dysfunctional Beliefs about Sleep Scale, Perceived Competence Scale about their sleep, and the RU SATED sleep health scale. Results: A two-step structural equation modeling strategy was used. Greater perfectionism discrepancies predicted greater dysfunctional beliefs about sleep (β = 0.45) and worse perceived sleep competence (β = −0.33). Moreover, greater dysfunctional beliefs and worse perceived sleep competence predicted worse sleep health (β = −0.23 and 0.59, respectively). Dysfunctional beliefs and perceived sleep competence significantly mediated the effect of maladaptive perfectionism on sleep health (β = −0.302). Discussion: Dysfunctional beliefs and sleep competence emerged as mechanisms through which maladaptive perfectionism may function as a barrier to healthy sleep. Although prior research positions perfectionism as a primary correlate of poor sleep, the current study identifies the role of beliefs about sleep as the pathway from perfectionism to poorer sleep health. The results highlight the importance of addressing both maladaptive beliefs about sleep as well as beliefs about one’s own sleep competency with undergraduate students with higher maladaptive perfectionism.

## 1. Introduction

A key personality construct in psychological research is perfectionism—a complex, multidimensional construct that has generated an extensive amount of research interest (e.g., [1]). Although difficult to define, perfectionism is commonly conceptualized as having high standards for one’s own performance. Further investigation into this construct has revealed that perfectionism can be both adaptive and maladaptive [2]. Adaptive perfectionism refers to characteristics, such as orderliness, that foster personal growth and striving for excellence, traits that are often encouraged in academia and the workplace [3]. Maladaptive perfectionism, however, refers to worry about making mistakes and a perceived discrepancy between one’s self-expectations and actual performance [4]. Maladaptive perfectionism can lead to self-defeating behaviors, such as task avoidance and excessive checking. Perfectionism is consistently identified as a predisposing and perpetuating factor for a wide range of mental health conditions and disorders, including depression, anxiety, and eating disorders [5,6]. Given the unique cognitive, emotional, and physiological characteristics associated with perfectionism [7], perfection could have serious implications for a critical health behavior—our sleep. The current study examines the links between perfectionism and sleep health with the goal of identifying potential sleep-related beliefs as underlying mechanisms.

## 2. Perfectionism and Sleep

A growing body of research suggests that perfectionism is linked empirically to sleep. The majority of this research has examined the association between perfectionism and disordered sleep, mainly insomnia (e.g., see review by [8]). Initial evidence of this association circulated nearly 30 years ago, when researchers first concluded that perfectionism may serve as a predisposing factor for the development of persistent insomnia [9]. Specifically, Lundh and et al. [9] found that patients with persistent insomnia scored higher on measures of perfectionism than the general population. Similarly, another study replicated these results and found that individuals with chronic insomnia were more likely to endorse “maladaptive” perfectionism compared to healthy controls [10]. Further, longitudinal studies have provided evidence of perfectionism serving as a reliable predictor of sleep disturbance [11].

The majority of this literature has examined perfectionism in relation to disordered sleep (i.e., insomnia) and sleep disturbance through the use of self-reported measures such as the Insomnia Severity Index (ISI; [12]), Pittsburgh Sleep Quality Index (PSQI; [13]), or daily sleep diaries. Only one study to date has investigated sleep through objective measurement, specifically polysomnography (PSG; [14]). This study found that perfectionism is related to objective sleep disturbances via polysomnographic markers of poor sleep, including total sleep time, number of awakenings, and apnea index [14].

Although the associations between perfectionism and disordered sleep are valuable, less is known about the links between perfectionism and general sleep health. Traditionally, sleep research has adopted a medical frame of reference, focusing on the correlates and outcomes of clinically diagnosable, disordered sleep [15]. More recently, researchers have shifted to a more positive frame of reference, focusing on the entire range of sleep health in order to understand the mechanisms that promote sleep health. To our knowledge, no studies to date have examined the association between perfectionism and general sleep health.

## 3. Mechanisms Linking Perfectionism to Sleep

**Figure 1:**
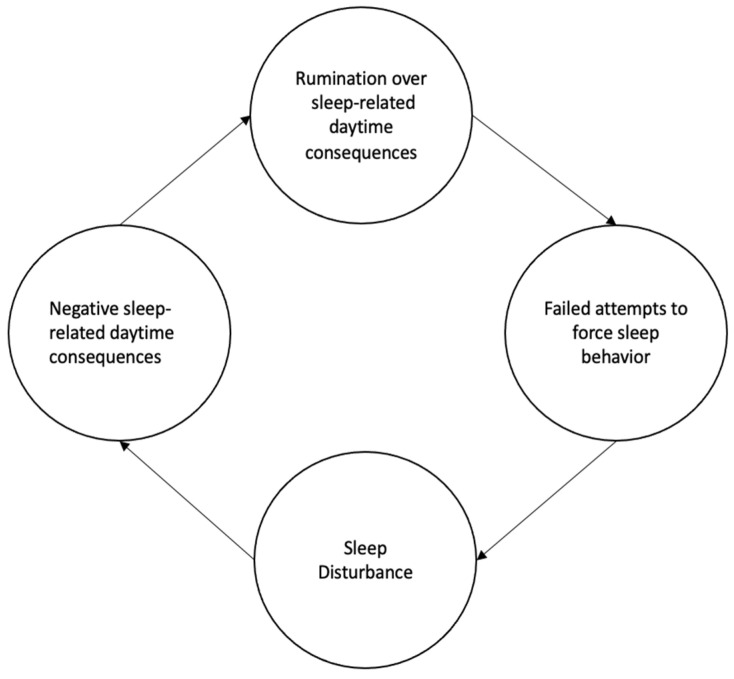
Conceptual model of the chronic cycle of rumination, unsuccessful sleep behavior attempts, sleep disturbance, and daytime consequences.

Perfection has been tied to sleep disturbance via cognitive, emotional, and physiological processes [1,14,16]. In particular, the internalization hypothesis highlights the role of rumination, a construct highly related to perfectionism, as a key contributor to sleep disturbance. Specifically, with perfectionism, excessive concern in general about making mistakes, worry, and unrealistic expectations for behavior contribute to physiological activation, which then interferes with sleep. This physiological activation contributes to hyperarousal (e.g., activation of the hypothalamic pituitary axis and increased release of cortisol into the bloodstream; [1,14]), resulting in attempts to regulate arousal through emotion regulation strategies. Maladaptive emotion regulation responses driven by perfectionism (e.g., worry, rumination) can foster experiences of anxiety and consequent sleep difficulties whereby the bed becomes associated with arousal instead of sleep behavior [1,14,17]. As a result of disrupted sleep, individuals then experience daytime consequences (e.g., sleepiness). As individuals high in perfectionism are concerned about daytime performance, performance-related distress can serve to perpetuate a chronic cycle of rumination over consequences of poor sleep on performance. This rumination then leads to disrupted sleep, which, in turn, provokes failed attempts to force sleep behavior, and, ultimately a return to negative daytime consequences ([9,14]; see Figure 1).

Although perfectionism has been identified as both a predisposing and perpetuating factor for sleep disturbance via emotional, cognitive, and physiological pathways [1], less is known about the direct ties between perfectionism and sleep-specific thoughts that are essential for healthy sleep. As such, it is important to identify the role of sleep-specific experiences as mechanisms tying perfectionism to sleep health. Dysfunctional beliefs about sleep and beliefs about one’s own sleep competency show potential as specific mechanisms tying perfectionism to sleep outcomes.

## 4. Sleep-Specific Mechanisms

### 4.1. Dysfunctional Beliefs

Beliefs about sleep and beliefs about one’s ability to sleep could be impacted by perfectionistic characteristics. First, characteristics of perfectionism such as high standards and a preference for order [2] could contribute to dysfunctional beliefs about sleep. Dysfunctional beliefs about sleep are a core component of models of insomnia (e.g., [18,19]). Compared with healthy sleepers, individuals with insomnia endorse higher levels of dysfunctional beliefs about the consequences of poor sleep, the causes of insomnia, and feelings of uncontrollability and helplessness related to sleep [20]. For instance, some individuals with insomnia fear the potential consequences of poor sleep on their daytime functioning, which then results in selective attention to and monitoring of any potential evidence of such consequences (i.e., changes in mood and energy levels). These dysfunctional beliefs are instrumental in heightening emotional arousal and distress, as well as in exacerbating sleep difficulties. As such, addressing the role of thoughts, attitudes, and beliefs related to sleep through incorporating cognitive therapy, for example, has become an essential piece of the treatment of sleep-related issues and disorders. To our knowledge, only one study to date has explored the mediating role of dysfunctional beliefs about sleep within the perfectionism–insomnia association [21]. Facets of multidimensional perfectionism and dysfunctional beliefs about sleep were significantly associated with insomnia symptoms. Further, dysfunctional sleep beliefs significantly mediated the associations between three perfectionistic dimensions—doubts about action, parental expectations, and parental criticism—and insomnia. Of note, this body of research focused exclusively on disordered sleep (insomnia), rather than the more inclusive continuum of sleep health.

### 4.2. Sleep Competence

Maladaptive perfectionism could be negatively tied to beliefs about one’s ability to sleep or sleep competence [2]. In general, perfectionists often exhibit lower beliefs in their competence or self-efficacy to achieve their very high personal standards [22]. Self-efficacy is an important predictor of action and is believed to affect an individual’s choice of activities, effort, outcome expectancies, and persistence [23]. Unfortunately, someone with maladaptive perfectionism may set unrealistic goals for their sleep and, accordingly, may exhibit lower sleep competence or efficacy to achieve these goals [8]. For example, an individual with high levels of maladaptive perfectionism may question their ability to engage in healthy sleep behaviors as they may hold exceedingly high expectations for those behaviors (e.g., “I don’t think I can fall into a deep sleep within 15 min,” “I cannot be exposed to *any* bright light in the evening”). One’s self-efficacy or perceived ability may suffer at the expense of setting high standards for sleep health behavior as a result of maladaptive perfectionism [24].

### 4.3. Consequence of Sleep-Specific Mechanisms

The consequences of maladaptive beliefs associated with perfectionism may be particularly dire for sleep. Specifically, the cognitive, emotional, and physiological activation that can accompany these maladaptive beliefs may not have time to dissipate if they occur during the bedtime period. Individuals with high levels of maladaptive perfectionism may be particularly at risk for rumination during the pre-sleep period, as they use this quiet, inactive time to reflect on their behavior and whether their own high standards were met [25,26,27]. Consequently, sleep health is a health behavior that is particularly vulnerable to the disruption of maladaptive beliefs about sleep and sleep competency [8].

### 4.4. Current Study Aims and Hypotheses

Perfectionism has been linked to disordered or disrupted sleep. Specifically, cognitive, emotional, and physiological processes appear to be activated in individuals with perfectionism with associated negative consequences for sleep [21,28]. Characteristics of maladaptive perfectionism, however, may be uniquely detrimental for sleep. In particular, unrealistic beliefs about sleep and low sleep competence could be associated with perfectionistic characteristics such as the need to maintain high standards of behavior and a preference for order. Although high standards could be seen as beneficial for health behaviors in general, sleep is unique in that efforts to force sleep have the undesired effect of undermining natural sleep processes. Consequently, an individual high in *maladaptive perfectionism* may set unrealistic goals for their sleep behavior based on *dysfunctional beliefs* about sleep, which, in turn, undermines their *sleep competency beliefs.* As a result, their overall *sleep health* may suffer. The present study investigated the sleep-related mechanisms of dysfunctional beliefs and sleep competency as potential links between maladaptive perfectionism and sleep health. Through this work, we can identify sleep-specific mechanisms that are essential for sleep health yet may be impacted by maladaptive perfectionistic tendencies. Furthermore, this study expands our understanding of the perfectionism-sleep association by assessing sleep health. As previously mentioned, prior research has focused on sleep from a disease-oriented framework, despite the importance of optimizing sleep even in the absence of disease [15]. Good, or healthy, sleep includes sleep dimensions such as regularity, satisfaction, alertness, timing, efficiency, and duration [15]. Examining sleep health allows for individuals’ sleep behaviors to be assessed along a broader, more inclusive spectrum than a disease model of sleep allows for.

Consequently, the specific aims of the current study were to: (1) examine the association between the specific dimensions of perfectionism and sleep health and (2) examine the role of sleep-related beliefs (dysfunctional beliefs about sleep and sleep competency) as mediators of the association between perfectionism and sleep health. Based on existing research, we hypothesized that greater maladaptive perfectionism would predict worse sleep health and this association would be mediated by greater dysfunctional beliefs about sleep, and worse sleep competency would underlie the association between perfectionism and sleep health.

## 5. Method

### Participants

Participants were 417 undergraduate students at a large, public university in the mid-Atlantic United States. Participants were recruited using SONA Systems, an online research participation pool for undergraduate students enrolled in one or more psychology courses at the present university, and they received extra credit in their psychology courses for participation. Participants self-selected the study, which was entitled “Psychological Variables and Sleep Health” from a list of available research studies. In order to participate, participants had to be 18 years or older and never diagnosed with a sleep disorder. Although not explicit inclusion criteria, given the nature of the SONA Systems research portal, participants were also undergraduate students who were enrolled in at least one Psychology course. After providing written consent, eligible participants were asked to complete a survey on Qualtrics, an online survey platform. Measures appeared in a randomized order for each participant. Additional measures were included in the survey that were not a part of the current study. Data collection for this study occurred from February to May, 2020. In mid-March, the university closed its physical campus and switched entirely to virtual learning due to the COVID-19 pandemic. The majority of the sample (81.2%) participated after this switch to all-virtual learning.

The present study was approved by the Virginia Commonwealth University Institutional Review Board (27 January, 2020; HM20018380).

## 6. Measures

### 6.1. Demographics

Students selected the race/ethnicities with which they identified from the following options: Asian, Black/African American, white, Hispanic/Latinx, Native American, Pacific Islander, Other. Students who selected more than one option were coded as Multiracial. Additionally, students were asked to choose their gender identity from the following options: Man, Woman, Non-binary/Transgender, Other.

### 6.2. Perfectionism

Perfectionism was measured with the Almost Perfect Scale-Revised (APS-R; [2], see Table 1). The APS-R consists of 23 items on a scale from 1 (strongly disagree) to 7 (strongly agree). Items are divided into three subscales representing different dimensions of perfectionism: High Standards, Order, and Discrepancy, with the former two subscales representing adaptive perfectionism and the latter representing maladaptive perfectionism. Responses to each item within a subscale were summed, with higher numbers indicating greater perfectionism. Because the three subscales are seen as independent forms of perfectionism, the original authors suggest that researchers do not calculate a total score or reliability estimates for all 23 items [2]; the subscales yielded strong reliability in the current sample (α = 0.87–0.94).

### 6.3. Sleep-Related Cognitions

Sleep-related cognitions were measured with the Dysfunctional Beliefs about Sleep Scale-16 (DBAS-16; [20], see Table 1). The DBAS-16 consists of four subscales representing types of sleep-related cognitions: perceived consequences of insomnia, worry/helplessness about insomnia, sleep expectations, and medication. Participants responded to each item on a scale from 0 (*strongly disagree*) to 10 (*strongly agree*). Responses to each item within a subscale were averaged, with higher averages indicating greater dysfunctional beliefs about sleep. Although the total score yielded strong reliability (α = 0.86), the reliability estimates among subscales were more variable (α = 0.54–0.80), with sleep expectations and medication demonstrating poor internal reliability (α < 0.60).

### 6.4. Perceived Sleep-Related Competence

Perceived sleep-related competence was assessed using the Perceived Competence Scale (PCS; [29]). To use this measure, researchers must insert the health behavior that they are examining into each item. In the current study, “healthy sleep habits” was inserted as the target behavior; however, participants may not be familiar with what constitutes healthy sleep. Therefore, a message providing examples of healthy sleep habits (limiting daytime naps to 30 min, avoiding stimulants such as caffeine or nicotine close to bedtime, establishing a regular relaxing bedtime routine, etc.) preceded administration of the PCS.

Participants indicated their level of agreement with four items on the PCS (e.g., “I feel confident in my ability to practice healthy sleep habits”) on a seven-point scale (1 = *Not at all true* to 7 = *Very true*). A total sum ranging from one to 28 was derived, with higher scores indicating higher perceived sleep competence. The PCS demonstrated strong reliability in the current sample (α = 0.93).

### 6.5. Sleep Health

The RU SATED scale assesses six dimensions of sleep health: regularity, satisfaction, alertness, timing, efficiency, and duration [13]. Participants respond to five items (“Are you satisfied with your sleep?”) on a scale ranging from 0 (*rarely*/*never*) to 2 (*usually*/*always*). A total score was summed representing participants’ overall sleep health, with higher scores indicating better sleep health. The RU SATED scale demonstrated poor internal reliability in the current sample (α = 0.62).

### 6.6. Attention Check

In order to assess for attention and random clicking, previous research suggests that inserting an instructional manipulation check, an item that appears similar in format to other items within the battery of measures but instructs the participant to select a particular option, successfully identified inattentive participant while not removing any true variance [30]. Therefore, three validation items appeared randomly throughout the battery. All attention check items featured five-point scales (1 = *Never*, 5 = *Always*). Participants were asked to either select “always,” “never,” or to continue without selecting any option.

### 6.7. Statistical Plan

**Figure 2:**
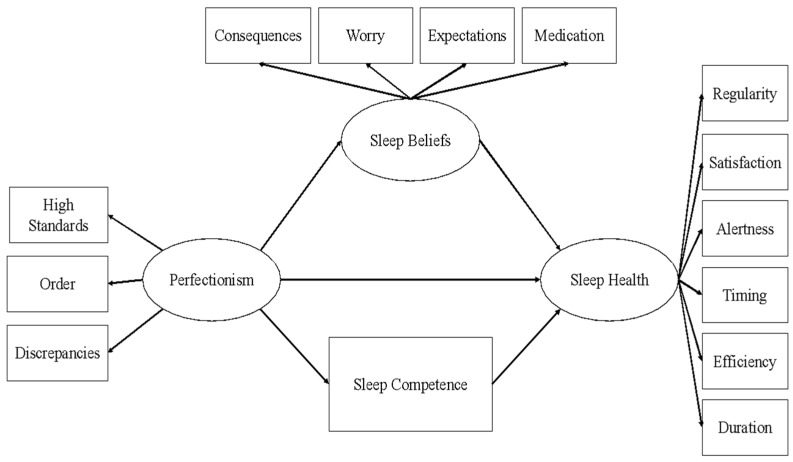
Structural equation model validating a hypothesized pattern of relationships among manifest and latent variables leading from perfectionism to sleep health.

All statistical analyses were conducted with SPSS version 26 and SPSS Amos 16.0. A structural equation model (SEM) was developed to validate a hypothesized pattern of relationships among manifest and latent variables leading from perfectionism to sleep health (see Figure 2). We hypothesized that greater perfectionism would be associated with greater dysfunctional beliefs about sleep, which would in turn be associated with worse sleep health. Additionally, we hypothesized that greater perfectionism would be associated with worse perceived sleep-related competence, which would in turn also be associated with worse sleep health. Thus, dysfunctional beliefs about sleep and perceived sleep-related competence served as parallel mediators in the association between perfectionism and sleep health. The three latent constructs in this model were perfectionism (comprising high standards, order, and discrepancies), dysfunctional beliefs about sleep (comprising consequences, worry, expectations, and medication), and sleep health (comprising regularity, satisfaction, alertness, timing, efficiency, and duration). Perceived sleep-related competence was entered as a manifest variable due to the brevity of the PCS.

A two-step structural equation modeling strategy was used. This strategy involves the separate estimation of the measurement model prior to the structural model, providing a comprehensive assessment of the full model. Model fit was assessed using goodness of fit indices (GFI, AGFI), relative fit indices (NFI, IFI, CFI), and the root mean squared error of approximation (RMSEA). Indices greater than 0.90 suggest adequate fit, and indices greater than 0.95 suggest good fit. An RMSEA of 0.08 or less suggests good fit [30].

Due to the unique social circumstances that coincided with data collection, it was necessary to assess the impact of the university’s closing of in-person learning due to COVID-19 on participant responses. A series of independent group *t*-tests were thus conducted to see if those who completed the survey prior to the university’s closing of in-person learning due to COVID-19 precautions significantly differed on any variable of interest from those who completed the survey after the university’s transition to virtual learning.

## 7. Results

### 7.1. Sample Characteristics and Data Cleaning

Participants who responded incorrectly to any validity items were removed from the sample (*n* = 25), as were participants who did not report any sleep data (*n* = 1). The final sample consisted of 383 participants. Participants had a mean age of 19.40 (*SD* = 2.19) and were mostly women (69.7%; see Table 2), closely approximating the gender demographics of the present university (62% women; [31]). The sample was racially diverse, with white (31.9%) and Black or African American (22.5%) students most commonly represented.

Across key variables, there was 9% missing data. Expectation maximization was used to address missing data. All key variables met assumptions of normality (skewness and kurtosis < 1.00). Descriptive statistics were calculated for all variables of interest (see Table 3). There were no significant differences on any variable of interest between those who completed the survey prior to implementation of university restrictions related to COVID-19 and those who completed the survey after these changes (see Table 4).

### 7.2. Structural Equation Modeling

The initial measurement model had poor fit, with all indices below the cutoff for adequate fit. Although all paths between manifest variables and their latent constructs were statistically significant (*p* < 0.001), several variables were loaded with low effect size (<0.40), including Standards and Order (subscales of perfectionism), Expectations (subscale of dysfunctional beliefs), and Alertness, Timing, and Efficiency (subscales of sleep health). Removing those manifest variables greatly improved model fit. The GFI, IFI, and CFI were in the range of good fit, at 0.97, 0.96, and 0.96, respectively. The AGFI, NFI, and RMSEA were in the adequate range, at 0.93, 0.94, and 0.07, respectively.

**Figure 3:**
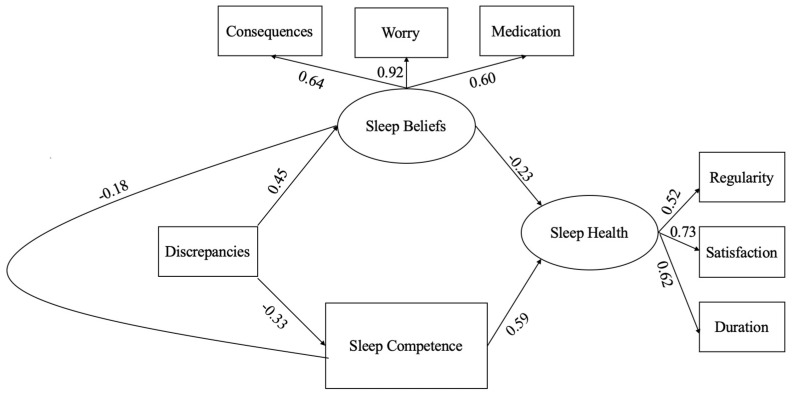
Final structural equation model with significant pathways presented.

As expected, the structural model also had adequate fit; however, the direct path between perfectionism and sleep health was not statistically significant. Moreover, modification indices suggested that correlating the error terms of dysfunctional beliefs about sleep and perceived sleep competence would improve model fit. In the final structural model, three fit indices suggested good fit and three suggested adequate fit (see Table 5). All pathways were statistically significant (*p* < 0.001; see Figure 3). Greater discrepancies predicted greater dysfunctional beliefs about sleep (β = 0.45) and worse perceived sleep competence (β = −0.33). Moreover, greater dysfunctional beliefs and worse perceived sleep competence predicted worse sleep health (β = −0.23 and 0.59, respectively). Dysfunctional beliefs and perceived sleep competence significantly mediated the effect of maladaptive perfectionism on sleep health (β = −0.302). Because the significance of the direct pathway between discrepancies and sleep health was reduced to non-significance once the mediators were introduced into the model, dysfunctional beliefs and perceived sleep competence fully mediated this association.

## 8. Discussion

As expected, both dysfunctional beliefs about sleep and perceived sleep competence surfaced as mediators in the association between perfectionism and sleep health. Specifically, within the present study, dysfunctional beliefs and sleep competence emerged as mechanisms through which maladaptive perfectionism may function as a barrier to healthy sleep. Furthermore, the parallel mediation by dysfunctional beliefs and sleep competence suggests that they both uniquely and simultaneously predicted sleep health. Although prior research positions perfectionism as a primary correlate of poor sleep [11,32], within the present sample perfectionism was not directly associated with sleep health in the presence of dysfunctional beliefs about sleep and sleep competency. This finding is consistent with research that suggests perfectionism is tied to sleep outcomes via cognitive, emotional, or physiological mechanisms [21,28,33].

In particular, discrepant, or maladaptive, perfectionism surfaced as a meaningful perfectionism factor associated with target variables, whereas adaptive facets of perfectionism were not associated directly or indirectly with sleep health. This finding suggests that adaptive perfectionism is neither beneficial nor detrimental for sleep health outcomes and is consistent with previous research that demonstrates that maladaptive perfectionism is more closely tied to sleep outcomes [10]. Higher levels of maladaptive perfectionism were associated with lower perceptions of sleep competence, which further contributed to poor sleep health outcomes. Previous research suggests that college students with higher levels of maladaptive perfectionism scored lower on general and social self-efficacy measures than both those with high levels of adaptive perfectionism and those with low levels of all forms of perfectionism [22]. Self-efficacy beliefs refer to an individual’s perception of their own ability to produce a desired outcome [23,34]. Sleep-specific self-efficacy, or sleep competence, would then refer to the extent to which an individual believes that their engagement in certain behaviors will produce desired sleep outcomes. Both general and domain specific sleep self-efficacy beliefs have been significantly, positively associated with sleep quality outcomes [35]. Indeed, some research even suggests that domain specific self-efficacy, such as sleep competency, is more important than general self-efficacy in producing behavioral outcomes [36,37]. As maladaptive perfectionism is defined as a mismatch between high standards for oneself and a low belief in one’s ability to achieve these standards; low self-efficacy in one’s ability to produce desired ideal sleep behavior outcomes appears to be a natural, reasonable progression.

Within the present study, greater maladaptive perfectionism was further associated with increased dysfunctional beliefs about sleep, which was then associated with worse sleep health overall. These findings are consistent with previous research showing dysfunctional beliefs about sleep serving as a mediator in the association between perfectionism and insomnia symptomatology [33]. Indeed, maladaptive perfectionism contributed to heightened occurrences of dysfunctional cognitions, which then contributed to worsened insomnia. The current study, however, extends this small body of research by examining the unique contributions of types of dysfunctional beliefs as a significant mechanism tying perfectionism to sleep health. Interestingly, dysfunctional beliefs about the need for medication, the daytime consequences of poor sleep, and general worries/feelings of helplessness related to sleep were significant mediators while expectations about sleep were not. Upon closer examination, the expectations factor is comprised of two items: “I need 8 h of sleep to feel refreshed and function well during the day” and “When I don’t get the proper amount of sleep on a given night, I need to catch up the next day by napping or the next night by sleeping longer.” It is possible that, for those who are endorsing maladaptive perfectionism, these statements may not fit with their ideal sleep behaviors. For example, the National Sleep Foundations sleep duration recommendations are 7–9 h per night for adults [38]. Furthermore, the recommendation to nap or “catch up” on missed sleep is counter to common sleep hygiene recommendations. Consequently, these expectations may not be endorsed by those who have high expectations for their sleep.

Additionally, the present study adds to research on perfectionism and sleep by investigating the role of sleep-specific beliefs in the context of sleep health, rather than sleep disturbance. Examining healthy sleep represents a shift from a deficit-based/treatment focus to a strength-based/promotion/prevention focus. In the present study, sleep health was operationalized as a multidimensional construct (regularity, satisfaction, alertness, timing, efficiency, and duration) that exists along a continuum. Consequently, we were able to examine predictors of optimal sleep, in addition to deficient sleep. Sleep-related beliefs mediated maladaptive perfectionism’s association with three of the six sleep health factors—regularity, satisfaction, and duration. Maladaptive perfectionism was associated with sleep health components of more irregular sleep timing, less satisfying sleep, and sleep of shorter duration. Alertness during the day, sleep efficiency, and the timing of sleep during the night (e.g., asleep between 2 and 4 am) were not tied to maladaptive perfectionism. Rather, sleep-related beliefs seem most relevant for feeling satisfied with one’s sleep, maintaining regular bed and wake times, and sufficient sleep duration. These findings reinforce the multiple facets of healthy sleep and the unique associations of maladaptive perfectionism with some, but not all, aspects of sleep.

## 9. Theoretical and Clinical Implications and Future Directions

The present findings have several important theoretical and clinical implications. First, although broader cognitive, emotional, and physiological mechanisms have been investigated as underlying the perfectionism-sleep association, the current study provides the first evidence for the role of multiple sleep-specific beliefs. Given the ruminative and perseverative characteristics associated with perfectionism, and the important role of sleep cognitions for healthy sleep, linking perfectionism with sleep-related beliefs is particularly relevant for understanding sleep in individuals with perfectionism. Cognitive–behavioral therapy for insomnia (CBTi) is the first line treatment for individuals with insomnia [39]. The current findings provide further information for how the cognitive component of CBTi could address dysfunctional beliefs about sleep and sleep competency concerns in individuals with maladaptive perfectionism. Furthermore, through the use of an SEM approach we uncovered which aspects of perfectionism, sleep-related beliefs, and sleep health are most pertinent. Future research could focus on the specific aspects identified in the current study as a target for further study or intervention efforts. Lastly, the examination of sleep health as an outcome provides further evidence for the need of multicomponent measures of sleep that address both optimal as well as impaired sleep health. Future research is needed to address how to manage sleep-related beliefs in individuals with maladaptive perfectionism in order to promote healthier sleep.

## 10. Limitations

There are several limitations in the current study. The use of an undergraduate college sample limits generalization to the broader population. Both perfectionism and unhealthy sleep are common concerns among college students given the inherent academic pressures and campus environment that they face [40,41]; therefore, their perfectionism and sleep may differ in some ways from other, adult samples. Moreover, the educational level of the sample (high school graduates, currently pursuing an undergraduate degree) and underrepresentation of men may limit the generalizability of the results. A strength of the current sample is its racial diversity, which is helpful for broader generalization and often lacking in perfectionism research.

Given the focus of prior research on emotional mechanisms, we did not investigate the role of anxious or depressive symptoms in the association between perfectionism and sleep. Future research could couple the role of sleep-related beliefs with their associated emotional consequences to further this line of research. The cross-sectional design of the current study precludes conclusions about directionality or causality. Future prospective or experimental research designs could help to isolate the temporal particulars of these associations. Additionally, all measures were self-reported. Actigraphy or sleep diaries may capture a more detailed, specific picture of sleep health and its associations with perfectionism.

The study data was collected during the COVID–19 pandemic. Although no differences were noted in study variables between students who completed the surveys pre- and during the pandemic, it is important to note that this was the social context for the majority of students.

## 11. Conclusions

The current study adds to existing knowledge tying perfectionism to sleep. Maladaptive perfectionism was indirectly tied to sleep health via sleep-related beliefs. The results suggest that maladaptive perfectionism is related to a continuum of sleep ranging from good to poor sleep. Furthermore, the results have implications for understanding how dysfunctional beliefs about sleep and beliefs about one’s own sleep competency can be linked to maladaptive perfectionism and predict better or worse sleep outcomes.

## Figures and Tables

**Table 1:** Subscale information for the APS-R and DBAS-16.

Scale	Sample Item	Number of Items	Range	Cronbach’s Alpha
*Almost Perfect Scale-Revised (Slaney et al., 2001)*				
Standards	“I have high expectations of myself.”	7	7–49	0.88
Order	“I am an orderly person.”	4	4–28	0.87
Discrepancies	“Doing my best never seems to be enough.”	12	12–84	0.94
Dysfunctional Beliefs about Sleep-16 (Morin et al., 2011)				
Consequences	“When I feel irritable, anxious, or depressed during the day, it is mostly because I did not sleep well the night before.”	5	0–10	0.73
Worry	“I am concerned that chronic insomnia may have serious consequences on my physical health.”	6	0–10	0.80
Expectations	“I need 8 h of sleep to feel refreshed and function well during the day.”	2	0–10	0.54
Medications	“I believe that insomnia is essentially the result of a chemical imbalance.”	3	0–10	0.59
Total Score		16	0–10	0.86

**Table 2:** Sample demographics.

	Frequency	Percentage
Gender Identity		
Man	106	27.7
Woman	267	69.7
Non-Binary/Transgender	9	2.3
Missing	1	0.3
Ethnicity		
Asian	70	18.3
Black/African American	86	22.5
White	122	31.9
Hispanic/Latinx	36	9.4
Prefer not to answer	8	2.1
Other	9	2.3
Multiethnic	50	13.1
Missing	2	0.5

**Table 3:** Descriptive statistics.

	Range	Mean	Standard Deviation
Perfectionism			
High Standards	7–49	39.84	7.31
Order	4–28	19.88	5.38
Discrepancies	12–84	50.86	17.44
Dysfunctional Beliefs			
Total Score	0–10	4.99	1.68
Consequence	0–10	5.18	2.13
Worry	0–10	4.29	2.22
Expectation	0–10	6.47	2.43
Medication	0–10	4.02	2.23
Sleep Competence	1–7	4.78	1.44
RU SATED-Global			
Total Score	0–12	6.92	2.51
Regularity	0–2	1.18	0.69
Satisfaction	0–2	0.95	0.66
Alertness	0–2	1.17	0.75
Timing	0–2	1.46	0.69
Efficiency	0–2	0.91	0.75
Duration	0–2	1.25	0.71

**Table 4:** Mean differences in participant responses before and after the transition to virtual learning due to COVID–19.

	Mean before COVID Transition (*n* = 67)	Mean after COVID Transition (*n* = 316)	*p*
Perfectionism	110.51	110.72	0.95
Dysfunctional Beliefs	5.02	4.98	0.88
Sleep Competence	4.86	4.76	0.61
Sleep Health	6.61	6.98	0.27

**Table 5:** Fit indices of final model.

Fit Index	Fit Statistic	Goodness-of-Fit
GFI	0.97	Good
AGFI	0.93	Adequate
NFI	0.94	Adequate
IFI	0.96	Good
CFI	0.96	Good
RMSEA	0.71	Adequate

## Data Availability

The data presented in this study are available upon reasonable request from the corresponding author.
